# Estrogen Reverses HDAC Inhibitor-Mediated Repression of *Aicda* and Class-Switching in Antibody and Autoantibody Responses by Downregulation of miR-26a

**DOI:** 10.3389/fimmu.2020.00491

**Published:** 2020-03-24

**Authors:** Paolo Casali, Tian Shen, Yijiang Xu, Zhifang Qiu, Daniel P. Chupp, John Im, Zhenming Xu, Hong Zan

**Affiliations:** Department of Microbiology, Immunology & Molecular Genetics, University of Texas Long School of Medicine, UT Health San Antonio, San Antonio, TX, United States

**Keywords:** *Aicda*, AID, antibody response, class switch DNA recombination, estrogen, estrogen receptor α, HDAC inhibitor, microRNA

## Abstract

Estrogen contributes to females' strong antibody response to microbial vaccines and proneness to autoimmunity, particularly antibody-mediated systemic autoimmunity, in females. We have hypothesized that this is due to estrogen-mediated potentiation of class switch DNA recombination (CSR) and somatic hypermutation (SHM). As we have shown, estrogen boosts AID expression, which is critical for both CSR and SHM, through upregulation of HoxC4, which together with NF-κB critically mediates *Aicda* (AID gene) promoter activation. We contend here that additional regulation of *Aicda* expression by estrogen occurs through epigenetic mechanisms. As we have shown, histone deacetylase inhibitors (HDIs) short-chain fatty acid (SCFA) butyrate and propionate as well as the pharmacologic HDI valproic acid upregulate miRNAs that silence AID expression, thereby modulating specific antibody responses in C57BL/6 mice and autoantibody responses in lupus-prone MRL/*Fas*^*lpr*/*lpr*^ mice. Here, using constitutive knockout *Esr1*^−/−^ mice and B cells as well as conditional knockout *Aicda*^*cre*/*cre*^*Esr1*^*flox*/*flox*^ mice and B cells, we showed that the HDI-mediated downregulation of *Aicda* expression as well as the maturation of antibody and autoantibody responses is reversed by estrogen and enhanced by deletion of ERα or E2 inhibition. Estrogen's reversion of HDI-mediated inhibition of *Aicda* and CSR in antibody and autoantibody responses occurred through downregulation of B cell miR-26a, which, as we showed, targets *Aicda* mRNA 3′UTR. miR-26a was significantly upregulated by HDIs. Accordingly, enforced expression of miR-26a reduced *Aicda* expression and CSR, while miR-26a-sponges (competitive inhibitors of miR-26a) increased *Aicda* expression and CSR. Thus, our findings show that estrogen reverses the HDI-mediated downregulation of AID expression and CSR through selective modulation of miR-26a. They also provide mechanistic insights into the immunomodulatory activity of this hormone and a proof-of-principle for using combined ER inhibitor-HDI as a potential therapeutic approach.

## Introduction

Estrogen plays an important role in boosting the production of mature antibodies and autoantibodies (1, 2). This provides an explanation for the greater antibody response to microbial vaccines and infections as well as the greater incidence of antibody-mediated autoimmunity, such as systemic lupus erythematosus, in females (3–17). Like protective antibodies against pathogenic microorganisms, pathogenic autoantibodies in humans and mice with systemic autoimmunity are generally class-switched and hypermutated, strongly suggest a role for estrogen in modulation of immunoglobulin (Ig) class switch DNA recombination (CSR) and somatic hypermutation (SHM). Ig CSR recombines S-S regions, generally Sμ with Sγ, Sα, or Sε, located upstream of exons of constant heavy-chain (C_H_), thereby encoding a new C_H_ region that endows an antibody with new biological effector functions (18). SHM introduces mainly point-mutations in Ig V(D)J segments, thereby providing the structural substrate for antigen-mediated selection of B cell mutants with higher affinity B cell receptors (BCRs). CSR and SHM are initiated by activation-induced cytidine deaminase (AID, encoded by *Aicda* in mice and *AICDA* in humans), which is expressed in B cells in a differentiation stage-specific fashion (19–21). As a potent DNA mutator, AID must be tightly regulated to prevent off-targeting effects, which can result in mutations in non-Ig genes, genomic instability, interchromosomal translocations and cellular neoplastic transformation (21).

Epigenetic mediators influence gene expression without modifying the genomic sequence. As we have suggested, such mediators, including DNA methylases, histone posttranslational modifiers, such as methyltransferases and acetyltransferases and non-coding RNAs, such as microRNAs (miRNAs), modulate B cell functions. They interact with genetic programs to regulate B cell functions, such as CSR, SHM and plasma cell differentiation, thereby informing the antibody response (1, 2, 22). We have shown that in addition to DNA methylation and histone acetylation in the *Aicda* locus, select miRNAs also provide an important mechanism for modulation of AID expression. miRNAs likely play important roles in B cell development, peripheral differentiation, and autoimmunity (2, 23–25). In B cells, miR-155, miR-181b, and miR-361 repress *Aicda* expression, while miR-30a and miR-125b repress *Prdm1* expression—*Prdm1* is the gene that encodes Blimp1, the plasma cell differentiation master transcription factor (23, 24). By binding to the evolutionarily conserved miRNA target sites in the 3′UTR of *Aicda* and *Prdm1* mRNAs, these miRNAs cause degradation of the mRNA transcripts and/or inhibit their translation (2, 26). As we have also shown, the expression of *Aicda-* or *Prdm1-*targeting miRNAs can be modulated by histone deacetylase (HDAC) inhibitors (HDIs), including short-chain fatty acids (SCFAs) butyrate and propionate, which alter histone acetylation in the host genes of these miRNAs (23–25). By altering chromatin accessibility, HDIs alter gene expression. As we demonstrated, HDI valproic acid, butyrate, and propionate epigenetic modifiers exert modulatory effects on intrinsic B cell functions even at moderate concentrations, thereby shaping effective antibody and autoantibody responses (23–25).

Estrone (E1), estradiol (E2), estriol (E3), and estetrol (E4) are the four major naturally occurring estrogens in females. E2 is the predominant estrogen in terms of both absolute circulating levels and activity, and, as we contend here, plays an important role in antibody and autoantibody responses. We have previously shown that estrogen activates AID by upregulating the homeodomain transcription factor HoxC4, which interacts with a conserved HoxC4/Oct binding site in the *AICDA/Aicda* promoter (21, 27, 28). At the transcriptional level, we have shown that estrogen-estrogen receptor (ER) complexes bind to three cooperative evolutionarily conserved estrogen response elements (EREs) in the *HOXC4/HoxC4* promoter and synergize with the signaling of CD154 or LPS and IL-4 to up-regulate HoxC4 expression, thereby inducing AID and CSR (28). ERs (ERα and ERβ, encoded by *Esr1* and *Esr2*, respectively) are ligand-dependent transcription factors that modulate gene transcription through recruitment to the target gene locus—ERα and ERβ may play different role in B cell differentiation and function (29). ER signaling can contribute to epigenetic changes (30). Indeed, estrogen is known to induce histone modifications, including histone acetylation, phosphorylation, and methylation, at the ER target gene promoters through dynamic interactions with histone modifying enzymes (30). Estrogen has been shown to suppress or stimulate miRNA expression and post-translationally regulate protein expression in human breast cancer cells, endometrial cells, mouse uterus cells, and rat mammary gland (31, 32). In ER^+^ breast cancer cells, estrogen reduces miR-26a expression (33).

Here, we addressed the role of estrogen in post-transcriptional regulation of AID expression and CSR in mouse B cells *in vivo* and *in vitro* in the presence of HDIs VPA, butyrate and propionate using *Esr1*^−/−^ mice/B cells, conditional knockout *Aicda*^*cre*^*Esr1*^*fl*/*fl*^ mice we generated by crossbreeding *Aicda*^*cre*^ mice with *Esr1*^*fl*/*fl*^ mice, as well as anti-estrogen drugs, including fulvestrant (a selective ER degrader, SERD) and Letrozole (an aromatase inhibitor that also inhibits endogenous estrogen synthesis). As epigenetic modifiers, SCFA HDIs inhibit *Aicda* expression and CSR through upregulation of select B cell miRNAs that silence *Aicda*, including miR-26a and miR-125a, in antibody and autoantibody responses (25). We found that estrogen abrogated the HDI-mediated inhibition of AID expression and CSR through ERα. Using *Aicda*^*cre*^*Esr1*^*fl*/*fl*^ mice and *Esr1*^−/−^ B cells, we showed that activated B cell-specific ERα deletion and SCFAs synergized to decrease AID expression and class-switched antibody responses. The combined impact of estrogen and VPA on class-switched antibody responses was assessed in normal mice antibody response and in the autoantibody response of lupus-prone MRL/*Fas*^*lpr*/*lpr*^ mice. Further, we analyzed how estrogen affected the role of HDIs as epigenetic modifiers, and found that ERα bound to ER-binding *cis*-elements in the miR-26a *CTDSPL* and *CTDSP1* host gene promoters, thereby inhibiting the expression of such a miRNA. Thus, estrogen/ERα provides an additional layer of epigenetic regulation of AID expression, as mediated by miR-26a that targets *Aicda* mRNA 3′UTR.

## Materials and Methods

### Mice

C57BL/6 (Stock No. 000664), *Esr1*^+/−^ (B6.129P2-*Esr1*^*tm*1*Ksk*^/J, Stock No. 004744) (34), MRL/*Fas*^*lpr*/*lpr*^ (MRL/MpJ-*Fas*^*lpr*^/J, Stock No. 000485) mice, and *Aicda-cre* transgenic *Aicda*^*cre*^ (B6; FVB-Tg(*Aicda-cre*)*1Rcas*/J, Stock No. 018422) (35) mice were purchased from the Jackson Laboratories. *Esr1*^*flox*/*flox*^ (*Esr1*^*fl*/*fl*^*, Esr1*^*tm*1.1*Gust*^) mice (36), which carry *loxP* sequences in the *Esr1* gene flanking exon 3 that encodes a conserved zinc finger type DNA binding domain, were obtained from Dr. J.-A. Gustafsson (Karolinska Institutet, Sweden). In BAC transgenic *Aicda*^*cre*^ mice, the bacterial *cre* recombinase gene was introduced in lieu of *Aicda* exon 1 in a supplementary *Aicda* locus and under the control of the *Aicda* promoter/enhancers within the BAC transgene (35). *Aicda*^*cre*^*Esr1*^+/+^ and *Aicda*^*cre*^*Esr1*^*fl*/*fl*^ mice were generated by crossbreeding *Aicda*^*cre*^ with *Esr1*^*fl*/*fl*^ mice. *Esr1*^−/−^ mice, and their *Esr1*^+/+^ littermates were generated by breeding *Esr1*^−/−^ mice. These mice were viable and fertile, and show no gross abnormalities. All mice were housed under pathogen-free conditions, and provided with autoclaved food and deionized water. The Institutional Animal Care and Use Committees of the University of Texas Health Science Center, San Antonio approved all animal protocols.

### SCFA HDAC Inhibitors, Estrogen, and Estrogen/ER Inhibitors

For *in vivo* studies, VPA sodium salt (VPA, Sigma-Aldrich) was dissolved in drinking water at 0.8% w/v (HDI-water). This yields a stable VPA serum level (400–600 μM) in mice, comparable to the serum concentration in humans under long-term VPA treatment (300–900 μM) (23, 37, 38). For SCFA treatment, sodium butyrate (140 mM, Sigma-Aldrich) and tributyrin emulsion (20.0 mg/ml), a prodrug of natural butyrate, which is a triglyceride containing three butyrate moieties that has low toxicity levels and is rapidly absorbed and hydrolyzed to butyrate by plasmatic esterases were added to the drinking waters (SCFA-water). Drinking water containing VPA or butyrate and tributyrin at the above concentration was always well-accepted by the mice. Exogenous estrogen E2 was made 1.0 μM by adding 40 mM E2 stock (dissolved in 100% ethanol) to drinking water. To inhibit ERα receptor and estrogen synthesis, some mice were injected subcutaneously every other day with fulvestrant (500 μg per injection) and letrozole (5 μg per injection). For *in vitro* experiments, VPA (250 or 500 μM), sodium butyrate (butyrate, 500 μM), sodium propionate (propionate, 2000 μM) and/or E2 (10, 30, or 100 nM) were directly diluted in culture medium.

### NP_16_-CGG Immunization and Titration of Total and NP-Binding IgM and IgG1

Female C57BL/6, *Esr1*^−/−^ and *Esr1*^+/+^ mice (8–12 weeks of age) were given water containing nil or VPA *ad libitum* 1 week before NP_16_-CGG (average 16 molecules of 4-hydroxy-3-nitrophenyl acetyl coupled to 1 molecule of chicken γ-globulin; Biosearch Technologies) immunization until end of the experiments. For NP_16_-CGG immunization, the mice were injected i.p. with 100 μg of NP_16_-CGG in 100 μl of alum (Imject® Alum, Pierce). Serum was collected 10 days later for titration of circulating total and NP-binding IgM and IgG1 using enzyme-linked immunosorbent assays (ELISAs), as we described (27, 39–41). Female *Aicda*^*cre*^*Esr1*^*fl*/*fl*^ and *Aicda*^*cre*^*Esr1*^+/+^ mice were given plain water or SCFA-water containing 140 mM sodium butyrate and 20.0 mg/ml tributyrin starting at the age of 5 weeks and i.p injected with NP_16_-CGG in 100 μl of alum at the age of 8 weeks. The mice were boost injected with NP_16_-CGG in PBS 3 weeks later. Serum total and NP_4_-binding IgM and IgG1 were measured by ELISA 7 days after boost injection.

### Mouse B Cells, CSR and Plasma Cell Differentiation

Naïve IgD^+^ B cells were isolated from 8-week-old C57BL/6 as described (23). B cells were resuspended in RPMI 1640 medium with 10% FBS (Hyclone), 50 mM β-mercaptoethanol and 1× antibiotic-antimycotic mixture (15240-062; Invitrogen) (FBS-RPMI) at 37°C in 48-well plates and stimulated with: LPS (5 μg/ml) from *Escherichia coli* (055:B5; Sigma-Aldrich) plus IL-4 (5 ng/ml; R&D Systems) for CSR to IgG1/IgE and plasma cell differentiation. Nil, VPA [250 or 500 μM; doses that were similar to serum concentrations of VPA-treated mice (42)], butyrate (500 μM) or propionate (2,000 μM) was then added to cultures and cells or supernatants were collected at various times.

### Flow Cytometry

For surface staining, B cells were reacted with PE-anti-B220 mAb (CD45R; RA3-6B2; eBioscience), PE-anti-CD19 (1D3; BD Biosciences), Alexa Fluor® 647-peanut agglutinin (PNA; Invitrogen), PE-anti-IgM mAb (AF6-78; BD Biosciences), FITC-anti-IgG1 mAb (A85-1; BD Biosciences), FITC-anti-IgG2a mAb (Clone R19-15; BD Biosciences).7-AAD, biotin-anti-CD138 mAb (281-2; BD Biosciences) followed by FITC-streptavidin (11-4317-87; eBioscience) or PE-streptavidin (12-4317-87; eBioscience). FACS analysis was performed on single cell suspensions. In all flow cytometry experiments, cells were appropriately gated on forward and side scattering to exclude dead cells and debris. Cell analyses were performed using a LSR-II flow cytometer (BD Biosciences), and data were analyzed using FlowJo software (TreeStar). All experiments were performed in triplicates.

### Luciferase 3′UTR Reporter Assays

The 3′UTRs of *Aicda* mRNA (NM 009645.2, National Center for Biotechnology Information) was PCR amplified using Phusion DNA polymerase (New England BioLabs.) from spleen B cell cDNA and cloned into the pMIR-REPORT miRNA Expression Reporter Vector System (Invitrogen), which allows for analysis of 3′UTR-mediated regulation of firefly luciferase activity. The mutant (mut) *Aicda* 3′UTR containing point mutations in the seed sequence of miR-26a target site was generated by PCR-based mutagenesis of the *Aicda* 3′UTR pMIR-REPORT vector. The sequence of constructs was confirmed by two independent sequencing reactions. Reporter constructs were cotransfected with the pRL-TK vector (Promega), which drives constitutive expression of *Renilla reniformis* luciferase, into mouse CH12F3 B cells by electroporation (250 V and 900 Ω) with a Gene Pulser II (Bio-Rad). Transfected CH12F3 B cells were then stimulated with CD154 (1 U/ml), IL-4 (5 ng/ml), and TGF-β (2 ng/ml) to induce AID expression and CSR to IgA) in the presence nil, E2 (30 nM), butyrate (500 μM) or butyrate (500 μM) plus E2 (30 nM) for 48 h. The abilities of butyrate and E2 to modulate reporter activity were determined by firefly luciferase activity and normalized to *Renilla* luciferase activity, according to the manufacturer's instructions, using the Luc-Pair™ Duo-Luciferase HS Assay Kit (GeneCopoeia).

### Quantitative RT-PCR (qRT-PCR) of mRNAs and miRNAs

For quantification of mRNA, pri-miRNA, germline I_H_-C_H_ transcripts, post-recombination Iμ-C_H_ transcripts and mature V_H_DJ_H_-C_H_ transcripts, RNA was extracted from 0.2 to 5 × 10^6^ cells using either Trizol® Reagent (Invitrogen) or RNesy Plus Mini Kit (Qiagen). Residual DNA was removed from the extracted RNA using gDNA eliminator columns (Qiagen). cDNA was synthesized from total RNA with the SuperScript™ III First-Strand Synthesis System (Invitrogen) using oligo-dT primer. Transcript expression was measured by qRT-PCR with the appropriate primers (Supplemental Table 1) using a Bio-Rad MyiQ™ Real-Time PCR Detection System (Bio-Rad Laboratories) to measure SYBR Green (IQ™ SYBR® Green Supermix, Bio-Rad Laboratories) incorporation with the following protocol: 95°C for 15 s, 40 cycles of 94°C for 10 s, 60°C for 30 s, 72°C for 30 s. Data acquisition was performed during 72°C extension step. Melting curve analysis was performed from 72 to 95°C. For quantification of mature miRNA transcripts, RNA was extracted from 0.5 to 5 × 10^6^ cells using miRNeasy® Mini Kit (Qiagen) and then reverse-transcribed with miScript II RT Kit (Qiagen) using the miScript HiSpec buffer. A Bio-Rad MyiQ™ Real-Time PCR Detection System was used to measure SYBR Green (miScript SYBR Green PCR Kit; Qiagen) incorporation according to manufacturer's instructions. Mature miRNA forward primers (Supplemental Table 1) were used at 250 nM in conjunction with the Qiagen miScript Universal Primer and normalized to expression of small nuclear/nucleolar RNAs Rnu6/RNU61/2, Snord61/SNORD61, Snord68/SNORD68, and Snord70/SNORD70. The ΔΔCt method was used for data analysis of qRT-PCR experiments.

### Chromatin Immunoprecipitation (ChIP) Assay

ChIP assays were performed as described (28). Briefly, B cells (5 × 10^6^) were treated with 1% formaldehyde for 10 min at 25°C to cross-link chromatin. After washing with cold PBS containing protease inhibitors (Roche Applied Science), chromatin was separated using nuclear lysis buffer (10 mm Tris-HCl, 1 mm EDTA, 0.5 m NaCl, 1% Triton X-100, 0.5% sodium deoxycholate, 0.5% Sarkosyl, pH 8.0) and resuspended in IP-1 buffer (20 mm Tris-HCl, 200 mm NaCl, 2 mm EDTA, 0.1% sodium deoxycholate, 0.1% SDS, protease inhibitors). Chromatin was sonicated to yield ~0.2–1.0-kb DNA fragments, precleared with agarose beads bearing protein G (Santa Cruz Biotechnology), and then incubated with rabbit mAbs to ERα (A4176, Abclonal) at 4°C. After overnight incubation, immune complexes were isolated using agarose beads bearing protein G, eluted with elution buffer (50 mm Tris-HCl, 0.5% SDS, 200 mm NaCl, 100 μg/ml Proteinase K, pH 8.0), and then incubated at 65°C overnight to reverse formaldehyde cross-links. DNA was extracted by phenol/chloroform and precipitated by ethanol and then resuspended in TE buffer (10 mm Tris-HCl, pH 8.0, 1 mm EDTA). The precipitated DNA was used as a template for qPCR analysis of *CTDSPL* and *CTDSP1* promoters using specific primers (Supplemental Table 1).

### Lupus Mice: Autoantibodies, Pathology and Disease

MRL/*Fas*^*lpr*/*lpr*^ mice were started on HDI-water *ad libitum* at 6- or 17-weeks of age, or were on untreated water throughout their life and scarified when moribund. Anti-nuclear antibody (ANA) and anti-dsDNA antibody titers were determined in sera. For ANA assays, sera were serially diluted in PBS (from 1:40 to 1:160), incubated on antinuclear Ab substrate slides (HEp-2 cell-coated slides, MBL-BION) and detected with a 1:1 mixture of FITC-anti-IgG1 and FITC-anti-IgG2a mAbs (R19-15; BD Biosciences). Images were acquired with a 40× objective on an Olympus CKX41 fluorescence microscope. Anti-dsDNA IgG and IgG2a antibody titers were measured in sera of MRL/*Fas*^*lpr*/*lpr*^ mice by ELISA as previously described (39). Titers were expressed in relative units (RUs), defined as the dilution factor needed to reach 50% of binding saturation and calculated using Prism? software (GraphPad Software, Inc.).

### Enforced Expression of miRNAs and miRNA Sponges

The miR-26a expression retroviral construct pMSCV-PIG-miR-26a-2 (43) was a gift from Joshua Mendell (Addgene plasmid # 64230), pMSCV PIG (Puro IRES GFP empty vector) was a gift from David Bartel (Addgene plasmid # 21654). The miR-26a-sponge retroviral construct pMG-miR26a-sponge was generated by insert a synthetic DNA sequence containing four miR-miR-26a complementary sites downstream of GFP in the pMG vector (Addgene plsasmid #58991), a MSCV based expression vector that also expresses GFP. with a 4-nt spacer between each site (44). A 3-nt mismatch region was placed in each microRNA binding site, because it has been shown to increase the effectiveness of sponge decoys. To generate the retrovirus, pMSCV-PIG-miR-26a-2, pMSCV PIG, pMG-miR26a-sponge or empty pMG vector together with the pCL-Eco retrovirus-packaging vector (Imgenex) were used to transfect HEK293T cells by a calcium phosphate-mediated transfection procedure (ProFection Mammalian Transfection System, Promega). Viral supernatants were harvested and used to transduce spleen B cells from C57BL/6 mice, as we reported (27, 40), after a 12 h LPS activation. Transduced B cells were then stimulated with LPS plus IL-4, in the presence of nil, E2 (30 nM), butyrate (500 μM), or butyrate (500 μM) plus E2 (30 nM) for 96 h before analysis of GFP^+^ and IgG1^+^ B cells by flow cytometry, as previously described (27, 40)—dead (7-AAD^+^) cells were excluded from analysis. Expression of AID and β-Actin proteins in transduced B cells were analyzed by immune-blotting (23) using rabbit anti-AID mAb (ZA001; Thermo Fisher Scientific) or anti–β-Actin mAb (AC-15; Sigma-Aldrich).

### Immunoblotting

B cells were lysed in Laemmli buffer. Cell extracts containing equal amounts of protein (20 μg) were fractionated through SDS-PAGE (10%). The fractionated proteins were transferred onto polyvinylidene difluoride membranes (Bio-Rad) overnight (30 V/90 mA) at 4°C. After blocking and overnight incubation at 4°C with anti-AID (ZA001; Invitrogen) or anti–β-Actin mAb (2F1-1, BioLegend), the membranes were incubated with HRP-conjugated secondary Abs. After washing with TBS–Tween 20 (0.05%), bound HRP-conjugated Abs were detected using Western Lightning Plus-ECL reagents (PerkinElmer Life and Analytical Sciences).

### Statistical Analyses

All statistical analyses were performed using Excel (Microsoft) or Prism® software. Differences in Ig titers, CSR and RNA transcript expression were analyzed with Student's paired (*in vitro*) and unpaired (*in vivo*) *t*-test assuming two-tailed distributions.

## Results

### HDI-Mediated Inhibition of the Class-Switched Antibody Response Is Reversed by Estrogen

**Figure 1:**
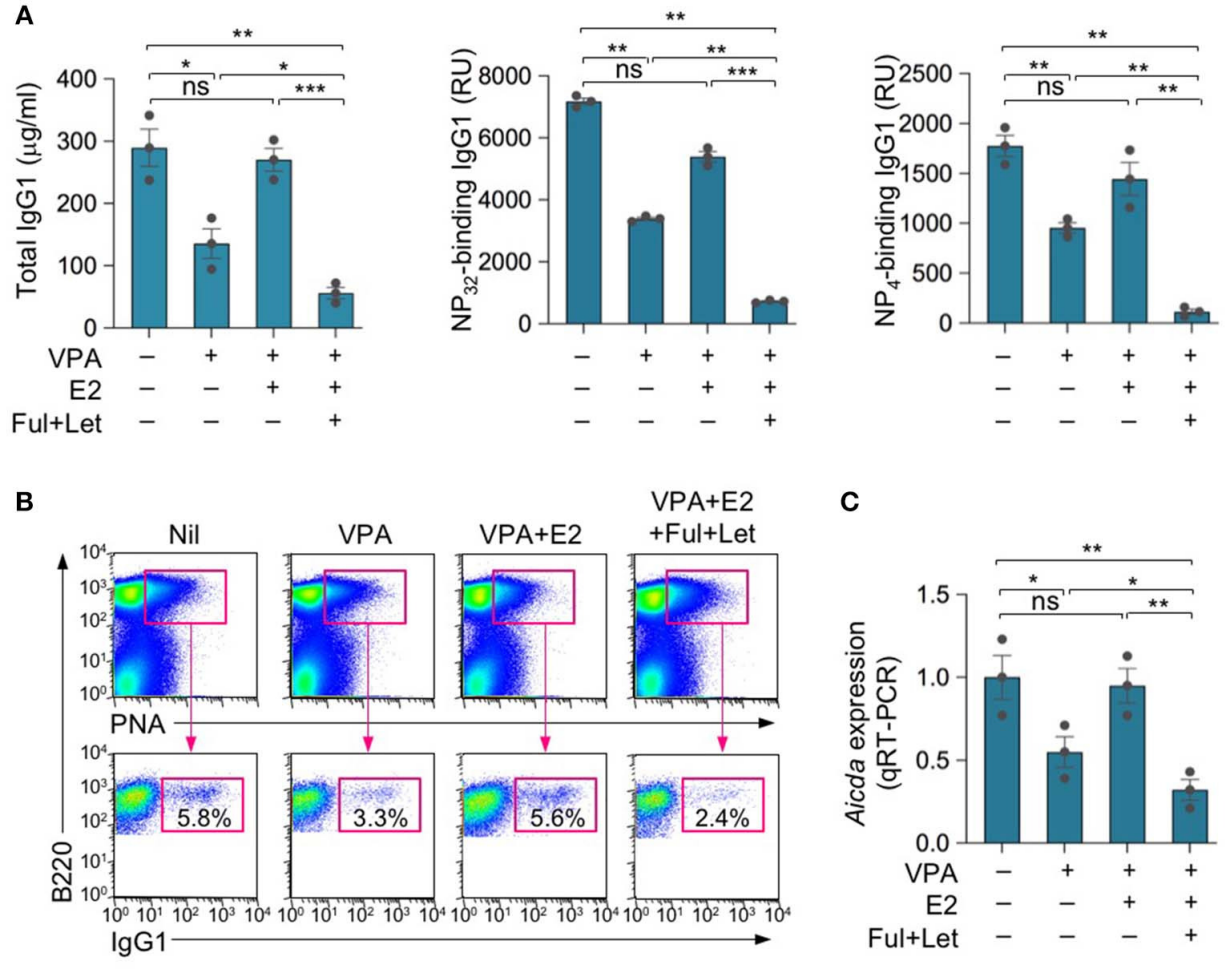
Estrogen reverses HDI-mediated inhibition of the antibody response. Female C57BL/6 mice were given plain or HDI-water starting at the age of 8 weeks and treated with nil, E2 (added to drinking water) or fulvestrant (Ful) and letrozole (Let) (i.p. injection) 1 day before injection with NP_16_-CGG. **(A)** Titers of NP_32_-binding IgG1 and high-affinity NP_4_-binding IgG1 (RU) in serum 10 days after NP_16_-CGG injection, as were measured by ELISA. Each symbol represents an individual mouse (*n* = 5). **(B)** Surface IgG1 expression in spleen B220^+^PNA^hi^ GC B cells 10 days after NP_16_-CGG injection. Data are representative of three independent experiments. Each symbol represents an individual mouse (*n* = 3). **(C)** Expression of *Aicda* transcripts in B220^+^ spleen B cells measured by real-time qRT-PCR. Expression was normalized to *Gapdh* expression and depicted as relative to the expression in B cells isolated from mice treated with nil, set as one. *p < 0.05, **p < 0.01, ***p < 0.001, ns, not significant (unpaired *t-*test). Data are mean ± SEM of three independent experiments.

We have shown that while HDIs, including the branched SCFA VPA and the four-carbon SCFA butyrate, dampen CSR/SHM and, therefore, the maturation of specific antibody responses, estrogen enhances CSR/SHM, thereby promoting maturation of antibody responses (23, 45). To define the impact of estrogen on HDI-mediated modulation of the antibody response, we treated female C57BL/6 mice with nil, HDI (in drinking water) only, HDI plus E2 (in drinking water) or HDI plus E2 (in drinking water), fulvestrant and letrozole (i.p. injection), and injected these mice with NP_16_-CGG, which preferentially induces IgG1 to NP, 1 day after the treatment was started. Consistent with our previous findings (23), HDI reduced the IgG1 response to NP, including total IgG1, NP_32_-binding and high-affinity NP_4_-binding IgG1 (Figure 1A). The HDI-mediated inhibition of NP-specific IgG1 production was reversed by E2, but greatly enhanced (in the presence of exogenous E2) by fulvestrant and letrozole. The reciprocal alteration of NP-specific IgG1 response by E2 or fulvestrant and letrozole was associated with a similar alteration in the proportion of IgG1^+^ PNA^hi^ germinal center B cells and *Aicda* expression (Figures 1B,C). Thus, consistent with our previous findings, SCFA HDIs inhibited *Aicda* expression, CSR and the antigen-specific class-switched antibody response (2, 23, 24). This inhibition was reversed by E2, but greatly enhanced by blocking ERα and inhibiting estrogen production.

### HDI-Mediated Inhibition of Autoantibody Response in Lupus MRL/*Fas^*lpr*/*lpr*^* Mice Is Reversed by Estrogen

**Figure 2:**
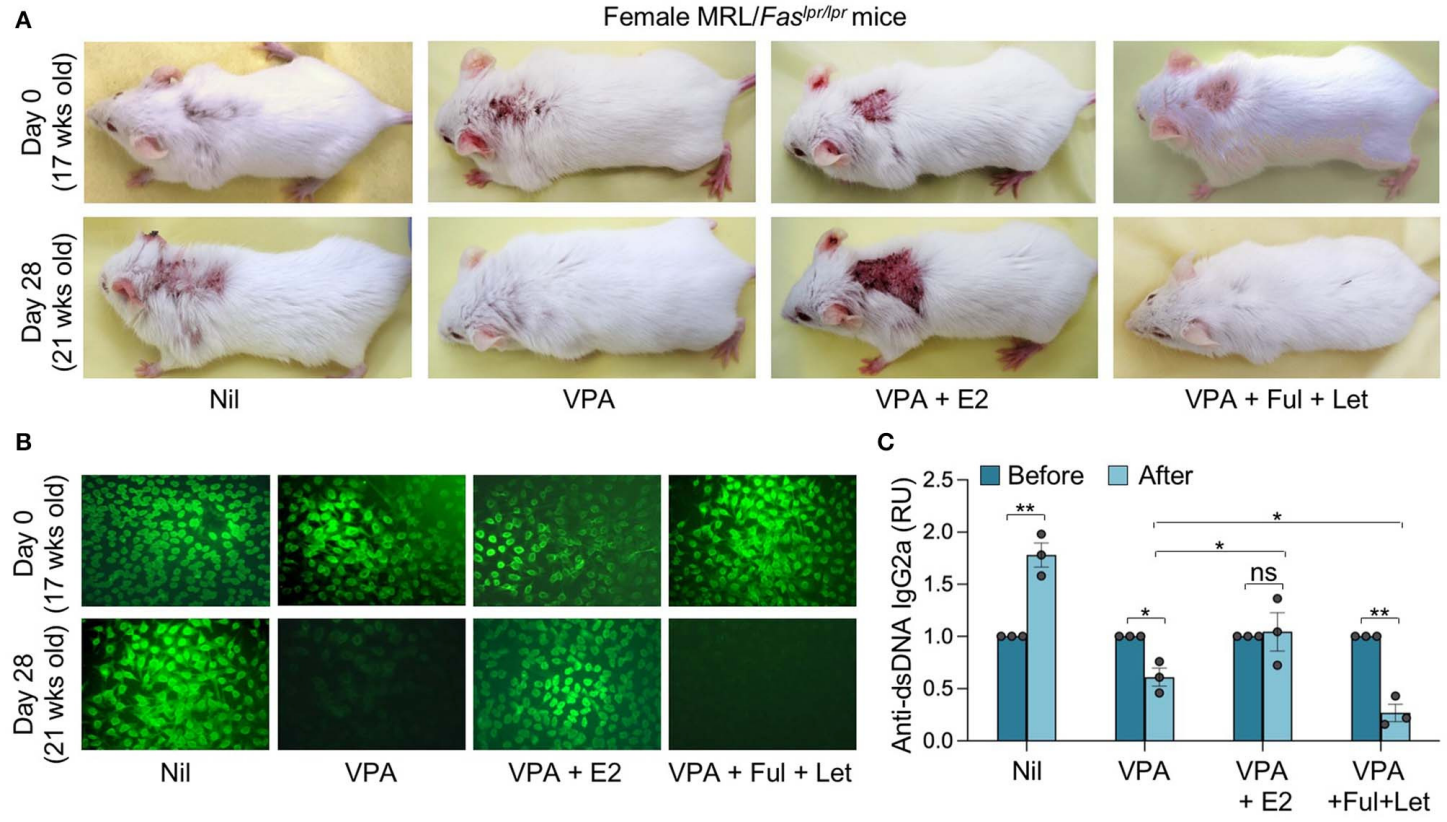
Estrogen reverses the HDI-mediated inhibition of skin lesions and autoantibody response in autoimmune MRL/*Fas*^*lpr*/*lpr*^ mice. Female MRL/*Fas*^*lpr*/*lpr*^ mice were treated with Nil, HDI (2% VPA in drinking water), HDI plus E2 or HDI plus fulvestrant (Ful) and letrozole (Let) (i.p. injection once every other day) starting at the age of 17 weeks and analyzed at the age of 21 weeks. **(A)** Dorsal images showing severe skin lesions in mice before (upper panels, 17 weeks of age) and after (lower panels, 21 weeks of age) 4 weeks of treatment. Data are representative of three independent experiments. **(B)** ANAs visualized by indirect immunofluorescence of HEp-2 cells that were incubated with sera (1:40 dilution) from MRL/*Fas*^*lpr*/*lpr*^ mice using FITC-labeled rat mAb to mouse IgG1 and IgG2a. Data are from one of three independent experiments yielding similar results. **(C)** Titers of circulating anti-dsDNA IgG2a before and after the treatment. *p < 0.05, **p < 0.01, ns, not significant (unpaired *t-*test). Data are mean ± SEM of three independent experiments.

**Figure 3:**
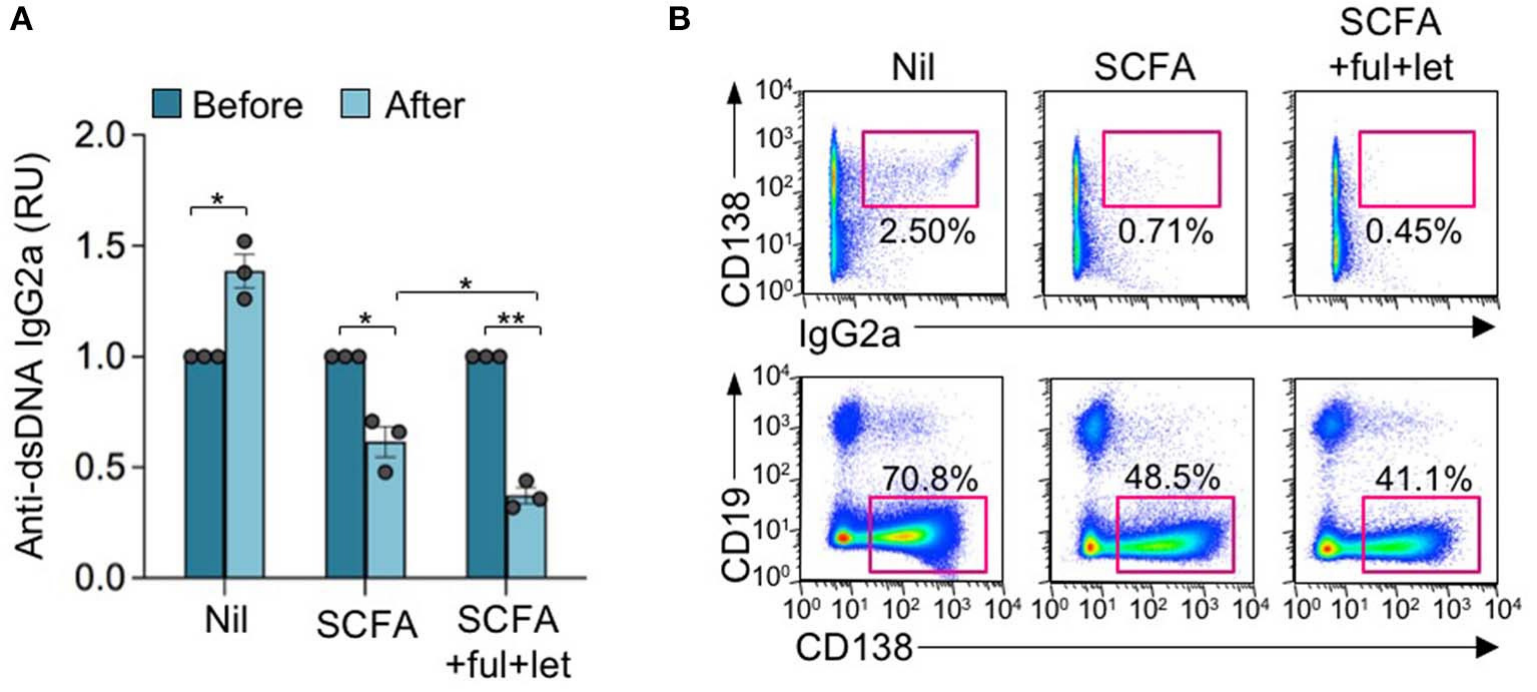
Estrogen reverses the SCFA HDI-mediated inhibition of autoantibody response in autoimmune MRL/*Fas*^*lpr*/*lpr*^ mice. Female MRL/*Fas*^*lpr*/*lpr*^ mice were treated with Nil, SCFA (140 mM sodium butyrate and 20.0 mg/ml tributyrin emulsion in drinking water), or SCFA plus fulvestrant (Ful) and letrozole (Let) (i.p. injection once every other day) starting at the age of 17 weeks and analyzed at the age of 21 weeks. **(A)** Titers of circulating anti-dsDNA IgG2a before and after the treatment. *p < 0.05, **p < 0.01. Data are mean ± SEM of three independent experiments. **(B)** CD138^+^IgG2a^+^ plasmablasts and CD19^−^CD138^+^ plasma cells in PBMCs, as analyzed by flow cytometry. Data are representative of three independent experiments.

Epigenetic dysregulation can result in aberrant antibody responses, such as those associated with autoimmunity or allergy, or neoplastic transformation. The pathogenesis of autoimmune diseases, including lupus, can be traced to both genetic susceptibility and epigenetic modifications arising from exposure to the environment, dietary intake, and hormonal factors (46). Consistent with our previous findings (23, 25), HDI also reduced autoantibody production and autoimmunity in lupus-prone mice, as reflected by reduced skin lesion, anti-nuclear antibody (ANA), and anti-dsDNA IgG2a titers in MRL/*Fas*^*lpr*/*lpr*^ mice treated with HDIs for 4 weeks (Figures 2A–C). Like the antigen-specific antibody response, the HDI-mediated inhibition of autoantibody response was reversed by E2, as shown by worse skin lesions and significantly increased ANA and anti-dsDNA IgG2a in MRL/*Fas*^*lpr*/*lpr*^ mice treated with HDI together with E2 as compared to the mice treated with HDI only. By contrast, the HDI-mediated attenuation of skin lesion and production of ANA and anti-dsDNA IgG2a autoantibody was enhanced by fulvestrant and letrozole (Figures 2A–C). Like VPA, SCFA butyrate reduced autoantibody production in MRL/*Fas*^*lpr*/*lpr*^ mice (Figure 3A). It also reduced IgG2a^+^CD138^+^ plasmablasts and CD19^−^CD138^+^ plasma cells in MRL/*Fas*^*lpr*/*lpr*^ mice (Figure 3B). As in VPA-treated mice, such SCFA butyrate-mediated reduction of IgG2a-producing B cells and plasma cells as well as autoantibodies was enhanced by fulvestrant and letrozole. Thus, in autoimmune lupus-prone mice, the HDI-mediated inhibition of the autoantibody response was impaired by estrogen but enhanced by blocking ER and reducing estrogen production.

### Activated B Cell-Specific ERα Deletion Synergizes With SCFA HDIs to Further Reduce the Class-Switched Antibody Response

**Figure 4:**
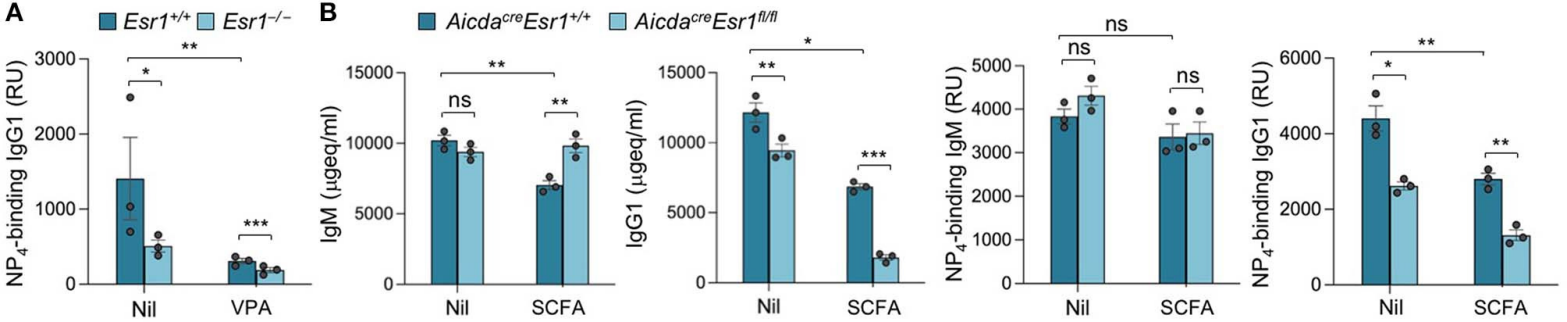
Activated B cell-specific ERα deletion and SCFA synergize to reduce class-switched antibody response. **(A)** Female *Esr1*^+/+^ and *Esr1*^−/−^ mice were given plain or HDI-water 1 day before injection with NP_16_-CGG. Titers of high-affinity NP_4_-binding IgG1 (RU) in serum 10 days after NP_16_-CGG injection, as measured by ELISA. Each symbol represents an individual mouse (*n* = 5). **(B)** Female *Aicda*^*cre*^*Esr1*^*fl*/*fl*^ and *Aicda*^*cre*^*Esr1*^+/+^ mice were given plain water (Nil) or SCFA-water containing 140 mM sodium butyrate and 20.0 mg/ml tributyrin starting at the age of 5 weeks, and i.p injected with NP_16_-CGG (in alum) at the age of 8 weeks. The mice were boost injected with NP_16_-CGG (in PBS) 3 weeks later. Circulating total and NP_4_-binding IgM and IgG1 were measured by ELISA 7 days after boost injection. Each symbol represents an individual mouse (*n* = 3 per group). Data are mean ± SEM of three independent experiments. *p < 0.05, **p < 0.01, ***p < 0.001, ns, not significant (unpaired *t-*test).

HDI-mediated inhibition of *Aicda* and CSR was significantly reversed by E2 in *Esr1*^+/+^ B cells but not in *Esr1*^−/−^ B cells—E2, HDI, or HDI plus E2 did not alter viability of either *Esr1*^+/+^ or *Esr1*^−/−^ B cells. Further, *Esr1* deletion, as in *Esr1*^−/−^ mice, also reduced the antigen-specific antibody response, as indicated by the reduced high affinity NP_4_-binding IgG1 in such mice immunized with NP_16_-CGG (Figure 4A). Consistent with our previous findings (23), HDI significantly decreased NP_4_-binding IgG1 in *Esr1*^+/+^ mice, and did so to antigen-specific IgG1 titers even lower in *Esr1*^−/−^ mice as compared to their *Esr1*^+/+^ littermates, likely due to lack of impact by endogenous estrogen. Thus, estrogen, through ERα, reverses HDI-mediated inhibition of *Aicda*, CSR and the antigen-specific antibody response.

To further define the role of B cell E2-ERα signaling in the modulation of SCFA HDI-mediated inhibition of the antibody response, we treated female *Aicda*^*cre*^*Esr1*^*fl*/*fl*^ and *Aicda*^*cre*^*Esr1*^+/+^ mice with SCFA HDI butyrate and immunized these mice with NP_16_-CGG. Consistent with the enhancing role of E2-ERα in *Aicda* expression and CSR (28), circulating total IgG1 and NP_4_-binding IgG1, but not IgM, were reduced in *Aicda*^*cre*^*Esr1*^*fl*/*fl*^ mice as compared to their *Aicda*^*cre*^*Esr1*^+/+^ counterparts (Figure 4B). Similar to the reduction in class-switched antibodies we showed in C57BL/6 mice and autoantibodies in lupus-prone MRL/*Fas*^*lpr*/*lpr*^ mice (Figures 1–3) (25), butyrate treatment reduced total and NP_4_-binding IgG1 in *Aicda*^*cre*^*Esr1*^+/+^ mice. Further, like in the HDI treated *Esr1*^−/−^ mice, the reduction of total and NP_4_-binding IgG1 by butyrate was even greater in *Aicda*^*cre*^*Esr1*^*fl*/*fl*^ mice, in spite of preservation of normal levels of total and NP_4_-binding IgM. Thus, B cell ERα deletion synergizes with SCFA to reduce the class-switched antibody response.

### Estrogen Reverses SCFA HDI Inhibition of *Aicda* Expression and CSR Through ERα

**Figure 5:**
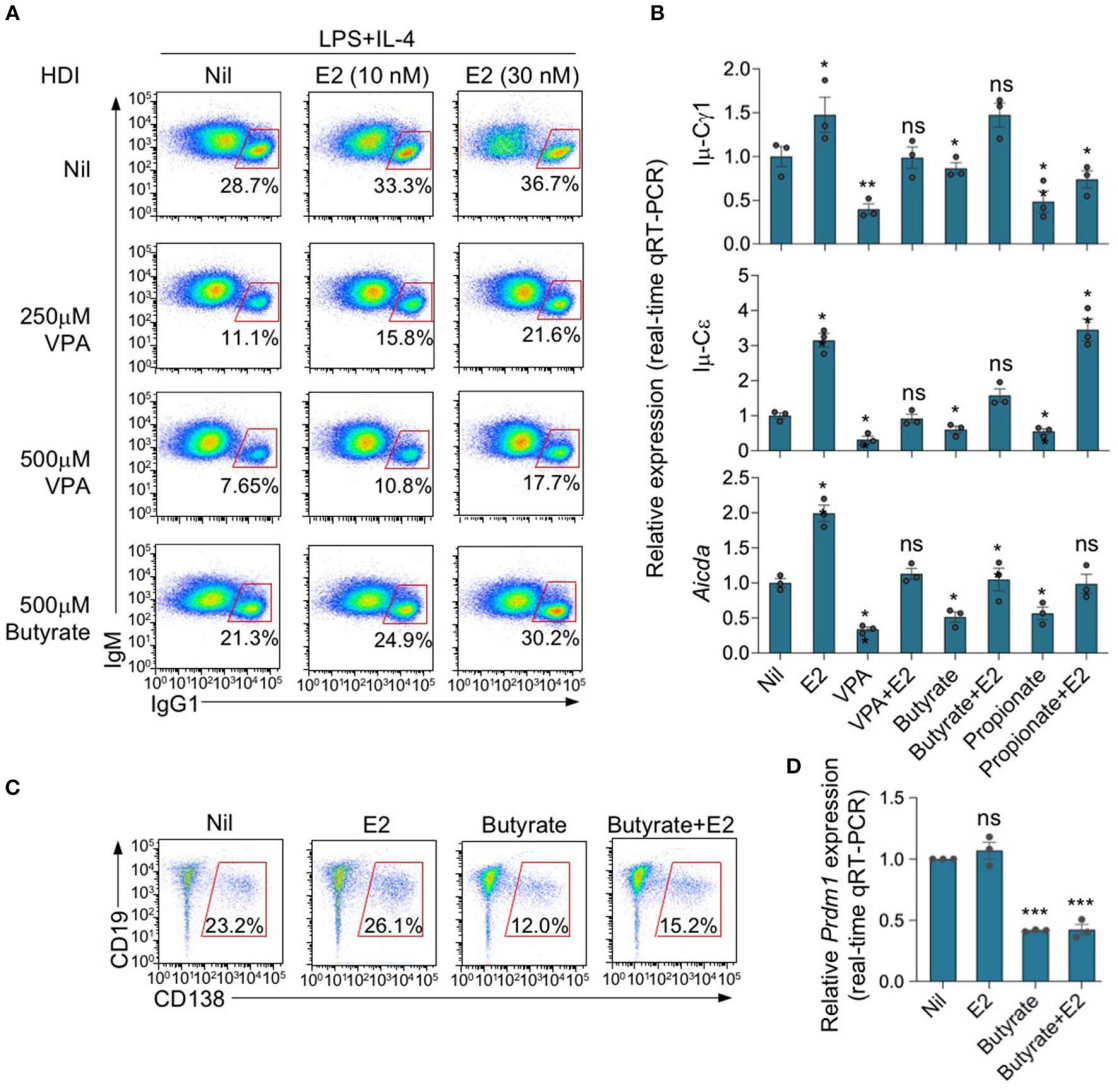
Estrogen impairs HDI mediated inhibition of *Aicda* expression and CSR. **(A)** Purified C57BL/6 B cells were stimulated with LPS plus IL-4, and treated with nil, VPA (250 or 500 μM) or butyrate (500 μM) in the presence of 0, 10, or 30 nM of E2. After 96 h of culture, the cells were analyzed for surface IgM and IgG1 by flow cytometry. Data are representative of three independent experiments. **(B)** Real-time qRT-PCR analysis of *Aicda* transcripts in B cells cultured for 60 h with LPS plus IL-4, in the presence of Nil, E2 (30 nM), VPA (500 μM), butyrate (500 μM), VPA (500 μM) plus E2 (30 nM) or butyrate (500 μM) plus E2 (30 nM). Expression was normalized to *Gapdh* expression and depicted as relative to the expression in LPS plus IL-4 stimulated B cells treated with nil, set as one. **(C,D)** Purified C57BL/6 B cells were stimulated with LPS plus IL-4, and treated with nil, E2 (30 nM), butyrate (500 μM) or butyrate (500 μM) plus E2 (30 nM). CD19^low^CD138^+^ plasma cells were analyzed by flow cytometry after 96 h of culture. Data are representative of three independent experiments **(C)**. Expression of *Prdm1* transcripts was analyzed after 60 h of culture by qRT-PCR, normalized to *Gapdh* expression and depicted as relative to that in the cells treated with nil, set as one **(D)**. *p < 0.05, **p < 0.01, ***p < 0.001, ns, not significant (unpaired *t*-test). Data are mean ± SEM of three independent experiments.

We have shown that HDIs inhibit antibody and autoantibody responses through inhibition of *Aicda* expression and CSR—as well as *Prdm1* expression and plasma cell differentiation (2, 23, 24). We have also shown that estrogen enhances *Aicda* induction and upregulated CSR (45). To assess the ability of E2 to reverse the SCFA-mediated inhibition of *Aicda* expression and CSR, we set up *in vitro* B cell stimulation cultures including nil or different doses of E2 (10 and 30 nM). The minimal amount of E2 contained in commercial FBS did not significantly affect the total amount of E2 in our cell culture medium—FBS from major vendors, such as Hyclone and ThermoFisher, contains 1.23–1.53 nM E2, which translates into 0.123–0.153 nM E2 in RPMI medium containing 10% FBS (47). As shown by a previous study of ours, E2 at concentration of <5 nM does not significantly alter AID expression and CSR (28). We, therefore, used RPMI medium containing 10% FBS in most *in vitro* experiments. Consistent with our previous findings, upon stimulation with LPS plus IL-4, HDI VPA, butyrate and propionate reduced CSR to IgG1 and IgE, as shown by reduced surface IgG1^+^ B cells and/or post-recombination Iμ-Cγ1 or Iμ-Cε transcripts (Figures 5A,B). Such CSR inhibition was associated with reduction of *Aicda* expression, as shown by qRT-PCR analysis, and was reversed by E2, which *per se* upregulated *Aicda* and CSR (Figure 5B). However, unlike *Aicda* expression and CSR, inhibition of *Prdm1* expression and plasma cell differentiation by HDI butyrate was not reversed by E2 (Figures 5C,D). Thus, estrogen impairs SCFA HDI-mediated inhibition of *Aicda* expression and CSR, thereby restoring CSR to pre-inhibition levels.

**Figure 6:**
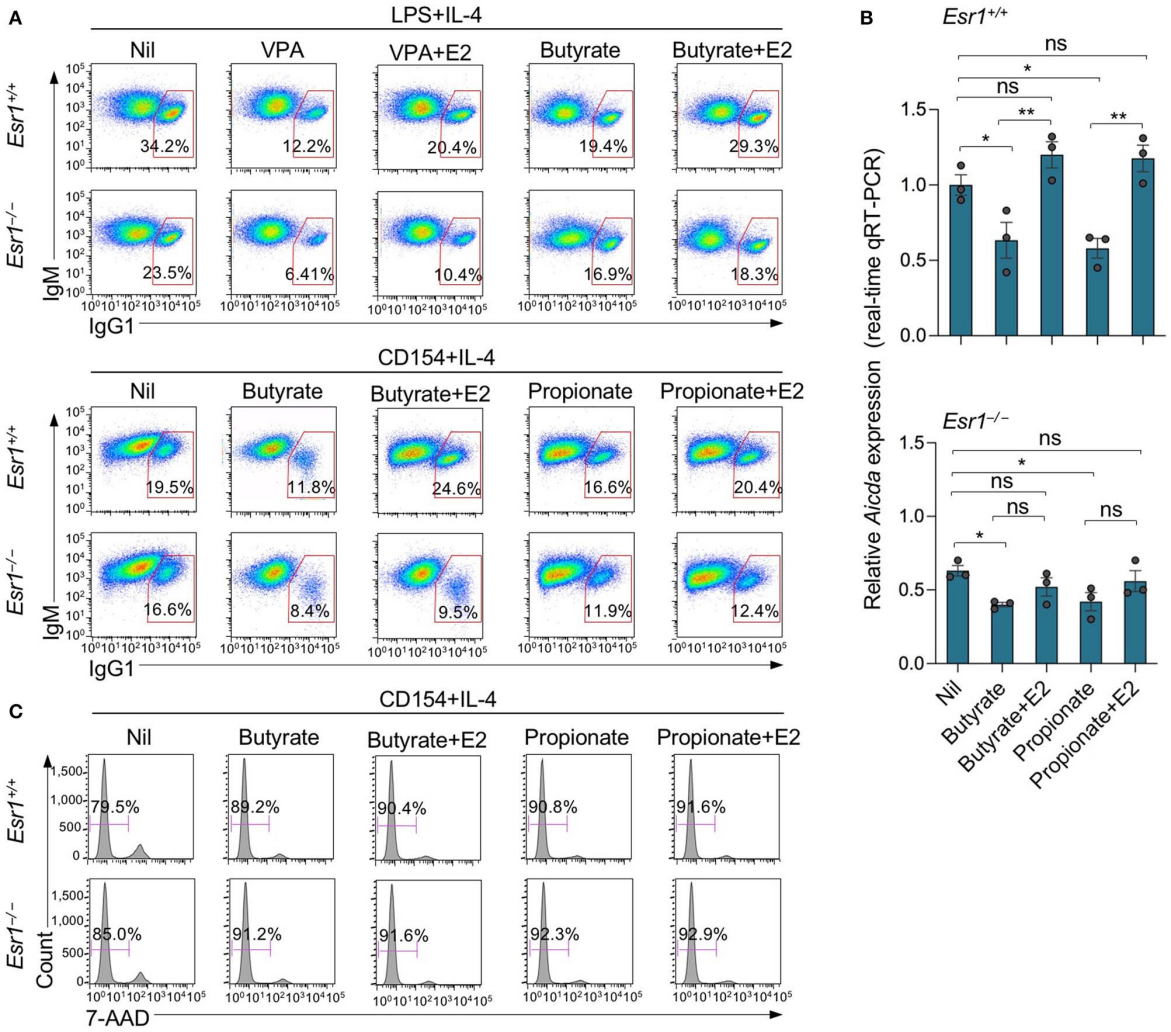
Estrogen impairs HDI mediated inhibition of *Aicda* expression and CSR through ERα. *Esr1*^+/+^ or *Esr1*^−/−^ B cells were stimulated with LPS plus IL-4 or CD154 plus IL-4 and treated with Nil, E2 (30 nM), butyrate (500 μM), propionate (2 mM), butyrate (500 μM) plus E2 (30 nM) or propionate (2,000 μM) plus E2 (30 nM). **(A)** After 96 h of culture with LPS plus IL-4 or CD154 plus IL-4, the cells were analyzed for surface B220 and IgG1 by flow cytometry. **(B)** After 60 h of culture with CD154 plus IL-4, *Aicda* transcripts were analyzed by real-time qRT-PCR. *p < 0.05, **p < 0.01, ns, not significant (unpaired *t-*test). Data are mean ± SEM of three independent experiments. **(C)** After 96 h of culture with CD154 plus IL-4, live (7-AAD^−^) cells were analyzed by flow cytometry. Data are representative of three independent experiments.

SCFA HDIs butyrate and propionate also inhibit *Aicda* expression and CSR in B cells stimulated by LPS plus IL-4 or CD154 plus IL-4 (25). In these B cells, the inhibition of *Aicda* expression and CSR was also reversed by E2 (Figures 6A,B). The E2-mediated reversion of SCFA HDI-mediated inhibition of *Aicda* expression and CSR was impaired by ERα deletion in *Esr1*^−/−^ B cells. In such *Esr1*^−/−^ B cells, *Aicda* expression and CSR to IgG1 were marginally lower than *Esr1*^+/+^ B cells, and were reduced to an even lower level by butyrate or propionate. *Esr1* knockout or treatment with butyrate and/or E2 did not alter cell viability, as shown by 7-AAD staining (Figure 6C). Thus, deletion of ERα as in *Esr1*^−/−^ B cells impairs the ability of estrogen to reverse the HDI-mediated inhibition of *Aicda* expression and CSR.

### Estrogen Reverses SCFA HDI-Mediated Upregulation of *Aicda*-Targeting miR-26a

**Figure 7:**
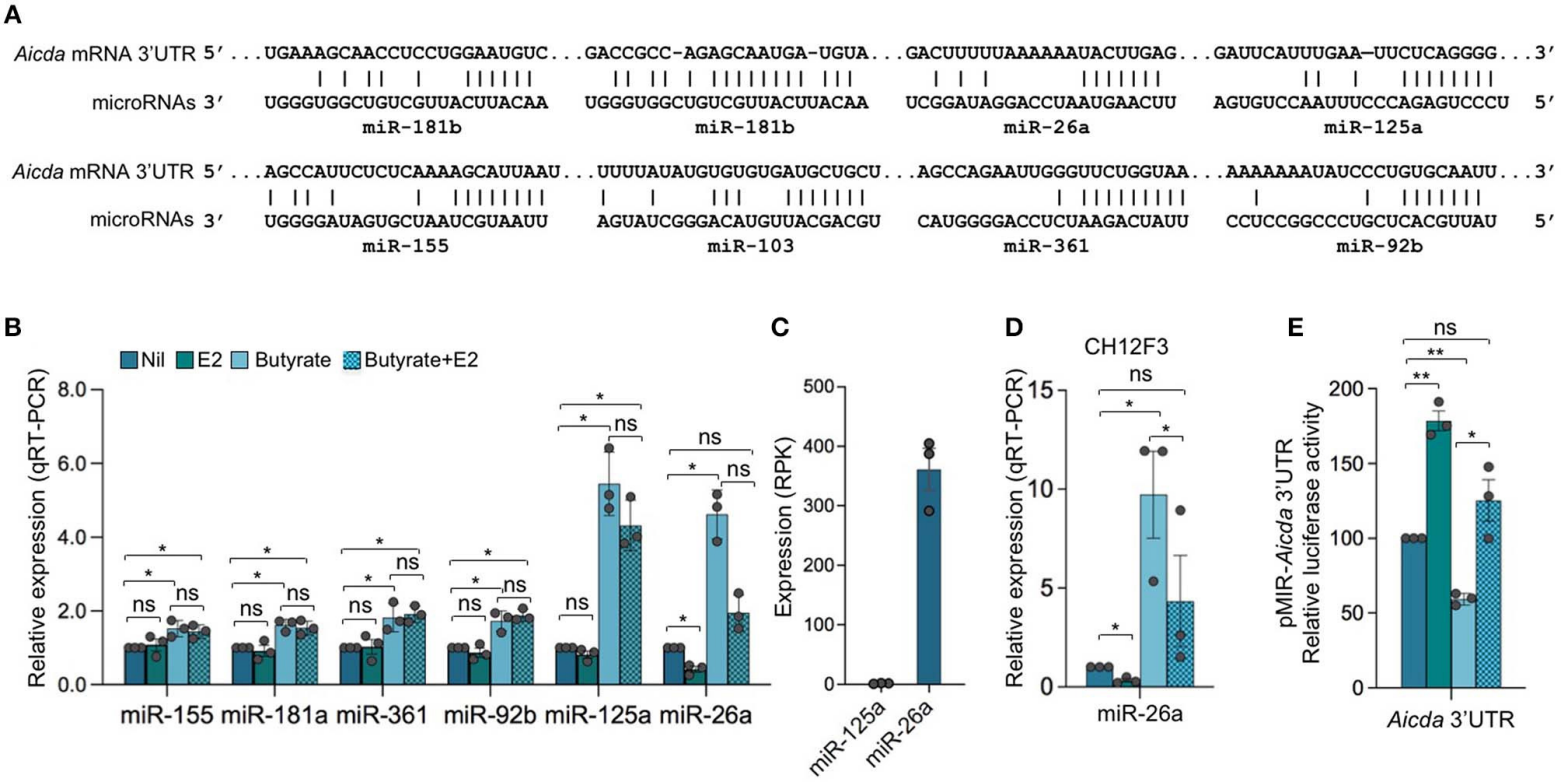
Estrogen impairs HDI mediated upregulation of *Aicda-*targeting miR-26a expression. **(A)** Alignment of miR-181b, miR-26a, miR-125a, miR-155, miR-103, miR-361, and miR-92b with their target sites in the 3′ UTR of *Aicda* mRNA (*Aicda* 3′UTR has two mR-181b target sites). **(B)** SCFA HDI butyrate upregulates *Aicda-*targeting miR-155, miR-181a, miR-361, miR-92b, miR-125a, and miR-26a, while estrogen downregulates miR-26a and reverses SCFA-mediated upregulation of this miRNA. B cells were stimulated with LPS plus IL-4 and treated with nil, E2 (30 nM), butyrate (500 μM) or butyrate (500 μM) plus E2 (30 nM) for 60 h. Expression of miRNAs was analyzed by qRT-PCR, normalized to expression of small nuclear/nucleolar RNAs Rnu6, Snord61, Snord68, and Snord70, and depicted as relative to the expression of this miRNA in B cells treated with nil, set as 1. Data are mean ± SEM from three independent experiments. **(C)** Expression of miR-125a and miR-26a in B cells stimulated with LPS plus IL-4 for 60 h, as analyzed by miRNA-Seq and depicted as RPK. Data are means ± SEM of three independent experiments. **(D)** miR-26a is upregulated by butyrate and downregulated by E2 in CH12F3 B cells. CH12F3 cells were stimulated with CD154 plus IL-4 and TGF-β, and treated with nil, E2 (30 nM), butyrate (500 μM) or butyrate (500 μM) plus E2 (30 nM) for 60 h. Expression of miRNA-26a was analyzed by qRT-PCR, normalized to expression of Snord70, and depicted as relative to the expression of this miRNA in CH12F3 cells treated with nil, set as 1. Data are mean ± SEM from three independent experiments. **(E)** SCFA and E2 modulate miRNAs that target *Aicda* 3′UTR. Luciferase activity in CH12F3 cells transfected with *Aicda* 3′UTRs (cloned into pMIR-REPORT luciferase reporter vector) after a 24-h treatment with nil, E2 (30 nM), butyrate (500 μM) or butyrate (500 μM) plus E2 (30 nM). Luciferase activity was measured 1.5 h after transfection. Transfection efficiency was controlled by normalizing to signal from co-transfected *R. reniformis* luciferase vector. Luciferase activity is depicted as relative to values in B cells cultured with nil, set as 100. Data are mean ± SEM from three independent experiments. *p < 0.05, **p < 0.01, ns, not significant (unpaired *t*-test).

As we showed by qRT-PCR and miRNA-Seq, HDI upregulate B cell miR-155, miR-181b, and miR-361, which silence AID by targeting *Aicda* 3′ UTR, as well as miR-125a, miR-92b, miR-26a, and miR-103, which, as we predicted, also target *Aicda* 3′ UTR (23, 24)—*Aicda* 3′UTR has two mR-181b target sites (Figure 7A). To define whether the expression of *Aicda*-targeting miRNA(s) can be modulated by estrogen in B cells induced to undergo CSR, we stimulated B cells with LPS plus IL-4 in the presence of nil, E2, butyrate or butyrate plus E2 for 60 h, before analyzing the expression of miRNAs. Consistent with our previous findings, miR-155, miR-181b, miR-361, miR-125a, miR-92b, and miR-26a were all upregulated by butyrate (Figure 7B). Among such miRNAs, however, miR-26a was the only highly expressed miRNA that was significantly reduced by E2, in the presence or absence of butyrate (Figure 7B)—miR-125a was also reduced by E2, but due to its low basal expression level, it did not likely play a significant role in the modulation of *Aicda* expression. As we have shown, miR-26a is highly expressed in B cells induced to undergo CSR and plasma cell differentiation, and it is one of the most abundant miRNAs expressed in B cells after stimulation by LPS plus IL-4 (24). In these B cells, the average RPKs of miR-26a, as measured by miRNA-Seq, was 418.2 (Figure 7C), which was more than 15 folds higher than the average RPMs of all the miRNAs analyzed (24).

To further define the role of miRNAs in SCFA- and E2-mediated modulation of *Aicda* expression, we generated luciferase reporter constructs containing *Aicda* 3′UTR. Such constructs were used to transfect mouse CH12F3 B cells that were induced to undergo CSR at a high rate. Like in primary B cells, in such cells, the *Aicda-*targeting miRNAs were upregulated by butyrate (25), and miR-26a was downregulated by E2 (Figure 7D). In CH12F3 B cells transfected with the *Aicda* 3′UTR reporter construct, the luciferase reporter activity was reduced by butyrate but increased by E2. Further, E2 reversed the butyrate-induced downregulation of luciferase reporter activity (Figure 7E).

**Figure 8:**
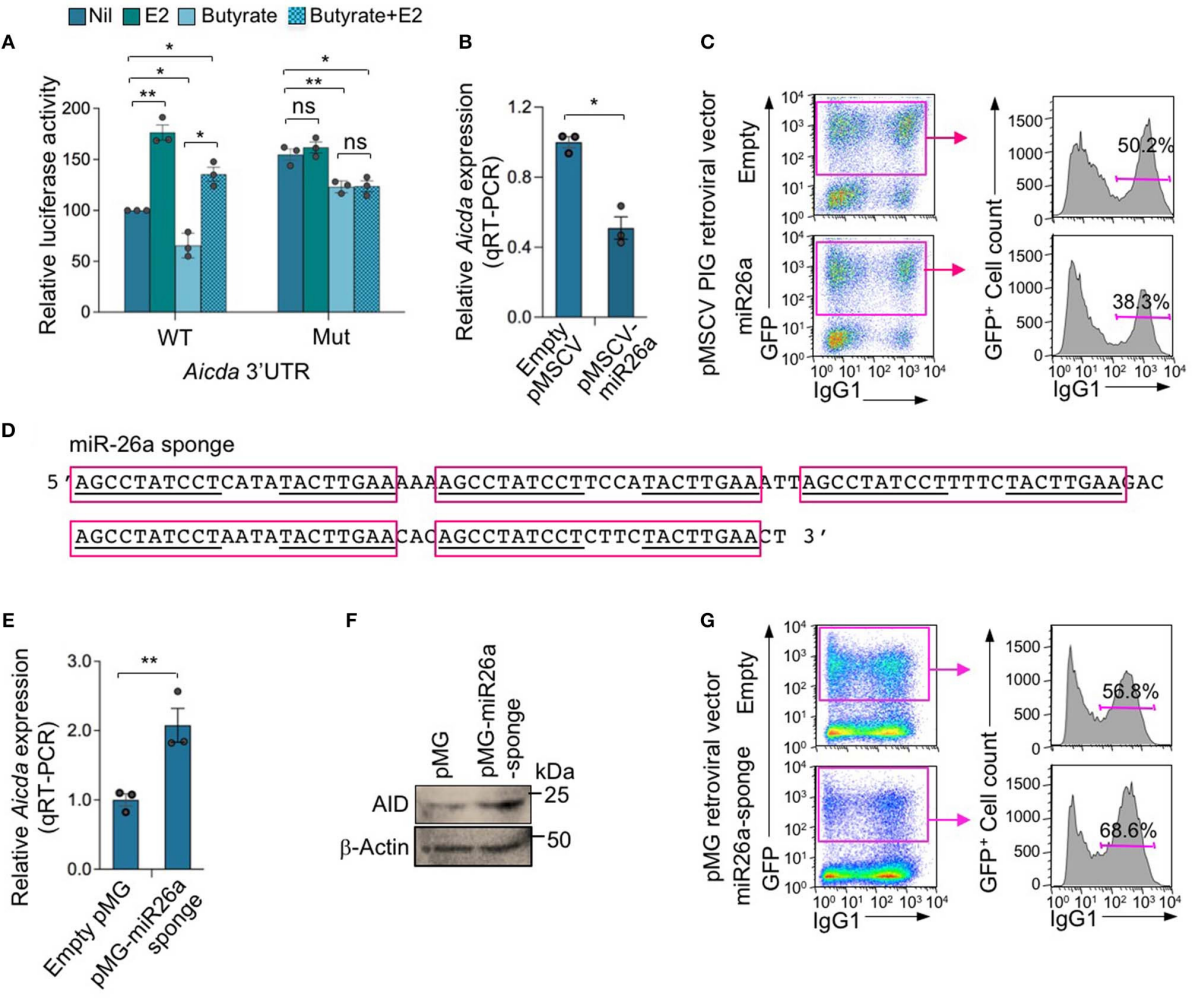
MIR-26a modulates *Aicda* expression and CSR. **(A)** Luciferase activity in CH12F3 cells transfected with wild-type or mutated *Aicda* 3′UTRs (cloned into pMIR-REPORT luciferase reporter vector) after a 24-h treatment with Nil, E2 (30 nM), butyrate (500 μM) or butyrate (500 μM) plus E2 (30 nM). Luciferase activity was measured 1.5 h after transfection. Transfection efficiency was controlled for by normalizing to signal from co-transfected *R. reniformis* luciferase vector. Luciferase activity is depicted as relative to values in B cells cultured with nil, set as 100. Data are mean ± SEM from three independent experiments. **(B)** Enforced expression of miR-26a reduced *Aicda*. B cells isolated from C57BL/6 mice were transduced with pMSCV-PIG-miR-26a-2 retroviral vector expressing GFP and miR-26a or empty pMSCV-PIG retroviral vector that expression GFP, and cultured for 48 h. Expression of *Aicda* was analyzed by real-time qRT-PCR, normalized to *Gapdh*, and depicted as relative to the expression of *Aicda* in B cells transduced with empty pMSCV-PIG retroviral vector, set as 1. Data are mean ± SEM from three independent experiments. **(C)** Enforced expression of miR-26a reduced CSR. Proportions of surface IgG1^+^ B cells among empty pMSCV-PIG or pMSCV-PIG-miR-26a-2 retroviral vector-transduced (B220^+^GFP^+^) B cells were analyzed by flow cytometry 96 h after transduction. Data are from one representative of three independent experiments. **(D)** Sequence of miR-26a sponge which contains five repeats of miRNA antisense binding sites (indicated by red boxes); sequences complimentary to mature miR-26a are underlined. **(E,F)** Expression of miR-26a sponge increased *Aicda* expression. B cells were transduced with pMG-miR-26a-sponge retroviral vector (expressing GFP and miR-26a sponge) or empty pMG retroviral vector (expressing GFP), and cultured for 48 h. Expression of *Aicda* was analyzed by real-time qRT-PCR, normalized to *Gapdh*, and depicted as relative to the expression of *Aicda* in B cells transduced with empty pMSCV-PIG retroviral vector, set as 1. Data are mean ± SEM from three independent experiments **(E)**. **(F)** AID and β-Actin proteins as analyzed by immune-blotting. **(G)** Expression of miR-26a sponge increased CSR. Proportions of surface IgG1^+^ B cells among empty pMG or pMG-miR-26a-sponge retroviral vector-transduced (B220^+^GFP^+^) B cells, as analyzed by flow cytometry 96 h after transduction. Data are from one representative of three independent experiments. *p < 0.05, **p < 0.01, ns, not significant (unpaired *t*-test).

To define whether miR-26a indeed directly targets *Aicda* 3′UTR to silence *Aicda* expression thereby reducing CSR, we constructed a luciferase reporter vector containing the *Aicda* 3′UTR sequence that was mutated to destroy the miR-26a target site. In CH12F3 B cells transfected with such a mutant *Aicda* 3′UTR reporter construct and cultured with CD154 plus TGF-β, the luciferase reporter activity was more than 60% higher than that in CH12F3 B cells transfected with the reporter construct including unmutated *Aicda* 3′UTR (Figure 8A). In fact, such luciferase reporter activity was comparable to that recorded in CH12F3 B cells transfected with the reporter construct including the unmutated *Aicda* 3′UTR and cultured with CD154 plus TGF-β in the presence of E2. Unlike in CH12F3 B cells transfected with unmutated *Aicda* 3′UTR luciferase reporter construct, in CH12F3 B cells transfected with the mutated *Aicda* 3′UTR luciferase reporter construct, the inhibition of luciferase reporter activity by butyrate was not reversed by E2. Thus, estrogen reverses SCFA HDI-mediated *Aicda* upregulation likely through downregulation of miR-26a which target *Aicda* 3′UTR.

### AID and CSR Are Inhibited by miR-26a Enforced Expression and Boosted by miR-26a Sponge

To further define the role of miR-26a in the modulation of *Aicda* expression and CSR, we enforced expressed this miRNA by transducing LPS-preactivated B cells with retroviral vector pMSCV-PIG-miR-26a-2 (43) expressing GFP and miR-26a or the control pMSCV-PIG vector expressing GFP but not a miRNA. The transduced B cells were cultured with LPS plus IL-4 for 72 or 96 h, respectively, before analyzing CSR to IgG1. pMSCV-PIG-miR-26a-2 retroviral vector-transduced B cells showed a significant reduction in *Aicda* expression and CSR to IgG1 (*p* = 0.003) as compared to pMSCV-PIG retroviral vector-transduced B cells (Figures 8B,C).

To further demonstrate the role of miR-26a in the modulation of *Aicda* expression and CSR, we constructed a pMG retroviral vector expressing a miR-26a sponge, capable of depressing the miR-26a target. The miR-26a sponge expression vector was generated by inserting a DNA fragment containing five miR-26a binding sequences (miR-26a sponge, Figure 8D) into the pMG retroviral vector. In B cells transduced with pMG-miR26a-sponge retroviral vector, expression of the miR-26a sponge resulted in an increased expression *Aicda* transcript and AID protein, and consequent CSR (Figures 8E–G). Thus, miR-26a significantly modulates *Aicda* expression and CSR.

### ERα Is Recruited to the Promoter of miR-26a Host Genes *CTDSPL* and *CTDSP1*, Directly or Indirectly Through Multiple *cis*-Elements, to Modulates miR-26a Expression

**Figure 9:**
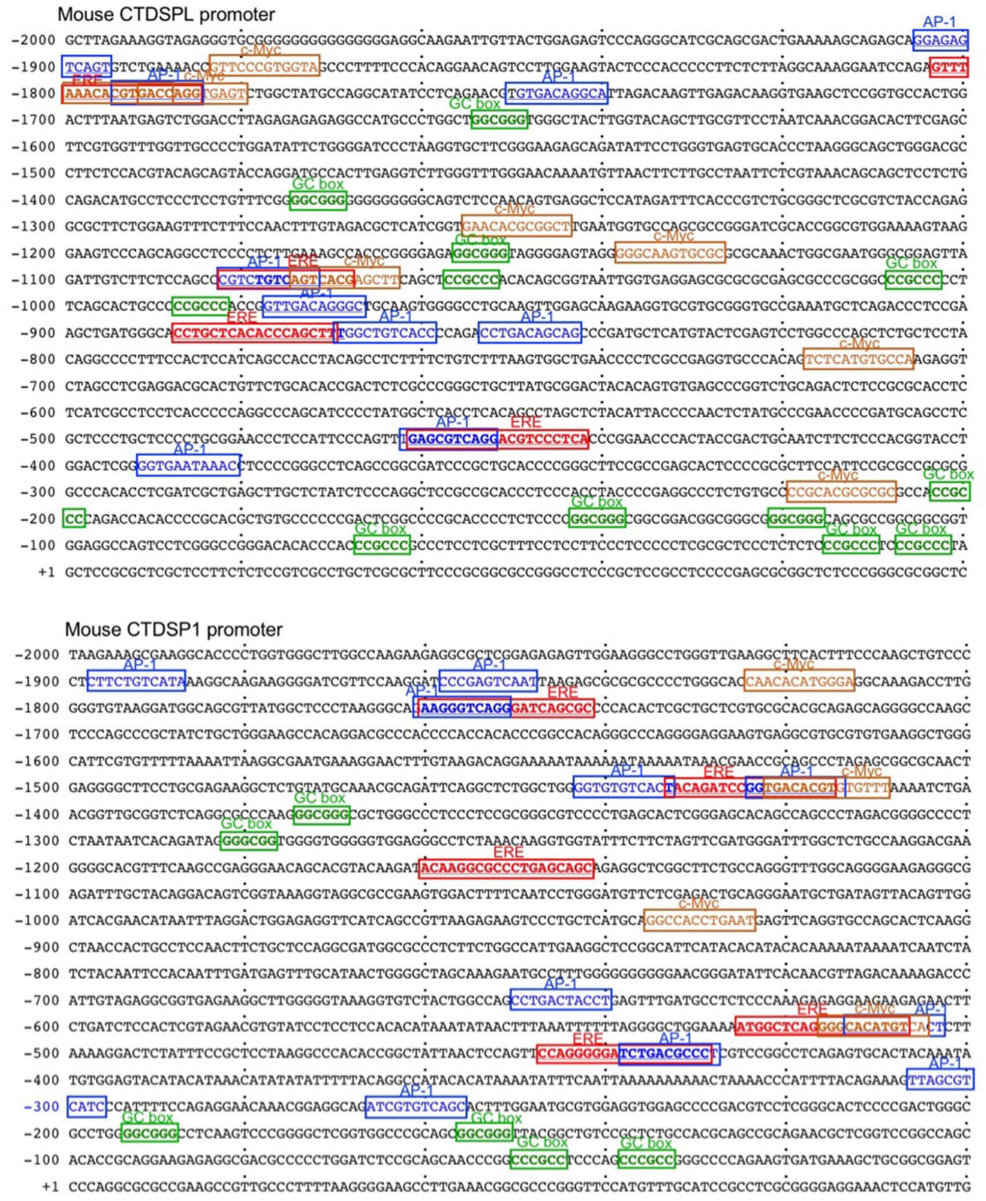
The promoters of *CTDSPL* and *CTDSP1* contain multiple putative EREs, and AP-1, Sp and c-Myc sites. The sequences of mouse *CTDSPL* and *CTDSP1* promoter regions. The EREs, AP-1 and c-Myc sites, as well as Sp1-binding GC box elements in the *CTDSPL* promoter and five EREs in the *CTDSP1* promoter are indicated.

**Figure 10:**
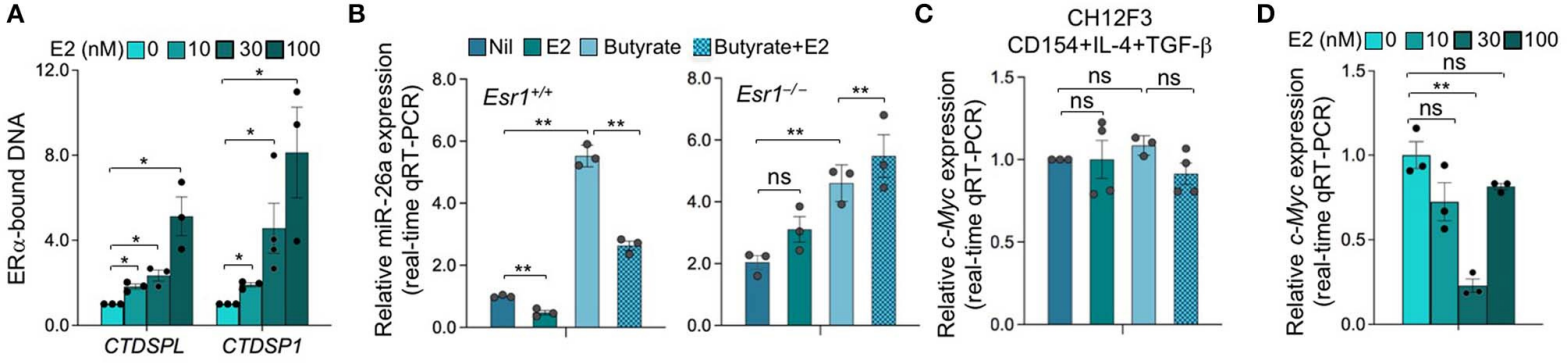
B cells miR-26a is regulated by ERα upon ERα recruitment to the promoters of miR-26a host genes *CTDSPL* and *CTDSP1* after E2 treatment. **(A)** Recruitment of ERα to the *CTDSPL* and *CTDSP1* promoters in B cells upon E2 treatment. B cells were stimulated with LPS plus IL-4 in the absence or presence of E2 (0, 30, and 100 nM) for 48 h, as analyzed by ChIP assay and qPCR and normalized to the input. Data are mean ± SEM from three independent experiments. **(B)** Estrogen downregulates miR-26a expression. *Esr1*^+/+^ and *Esr1*^−/−^ B cells were stimulated with LPS plus IL-4 and treated with nil, E2 (30 nM), butyrate (500 μM) or butyrate (500 μM) plus E2 (30 nM) for 60 h. Expression of miR-26a was analyzed by qRT-PCR, normalized to expression of small nuclear/nucleolar RNAs Rnu6, Snord61, Snord68, and Snord70, and depicted as relative to the expression of this miRNA in *Esr1*^+/+^ B cells treated with nil, set as 1. Data are mean ± SEM from three independent experiments. **(C)** CH12F3 cells were stimulated with CD154 plus IL-4 and TGF-β and treated with nil, E2 (30 nM), butyrate (500 μM) or butyrate (500 μM) plus E2 (30 nM) for 60 h. Expression of *c-Myc* transcripts was analyzed by qRT-PCR, normalized to expression of *Gapdh*, and depicted as relative to the expression of *c-Myc* in CH12F3 cells treated with nil, set as 1. Data are mean ± SEM from three independent experiments. **(D)** B cells were stimulated with LPS plus IL-4 in the absence or presence of E2 (0, 30, and 100 nM) for 60 h. Expression of *c-Myc* transcripts was analyzed by qRT-PCR, normalized to expression of *Gapdh*, and depicted as relative to the average expression level of *c-Myc* in B cells treated with nil, set as 1. Data are mean ± SEM from three independent experiments. *p < 0.05, **p < 0.01, ns, not significant (unpaired *t* test).

E2 can downregulate as many protein encoding genes and miRNA host genes as it upregulates (31, 32, 48). After binding to nuclear ERs, E2-bound ERs function primarily as transcription factors through a classical pathway by interacting specifically with EREs in the promoter of estrogen-responsive genes to elicit transcriptional activation or repression. E2-ER-dependent regulation of gene expression can also function through a non-classical pathways involving protein-protein interaction of DNA-bound ER with other DNA-bound transcriptions factors (49). E2-induced activation of complexes consisting of ER and Specificity protein (Sp) and Activating protein-1 (AP-1, c-Jun, and c-Fos) involves pathways that modulate expression of many genes (49–51). E2-ERαs can bind to DNA directly through ERE (or half ERE) or indirectly by association with an intermediary transcription factor such as AP-1 or Sp1. Transcription of genes that depend on AP-1 can be repressed by E2-ERs (52–55). E2 has been reported to repress gene transcription through ERα-Sp1, which recruits the transcriptional repressor-HDAC3 complex (56). We identified three and five EREs in the promoters of two host genes encoding miR-26a, *CTDSPL* and *CTDSP1*, respectively, as well as 10 AP-1 sites in each gene (Figure 9). Three AP-1 sites in the *CTDSPL* promoter and five AP-1 sites in the *CTDSP1* promoter overlap with EREs. We also identified 11 and 6 Sp1-binding GC box elements in promoter regions of *CTDSPL* and *CTDSP1*, respectively, further suggesting that E2-ERαs negatively regulate *CTDSPL* and *CTDSP1* expression. Accordingly, recruitment of ERα to both the *CTDSPL* and *CTDSP1* promoters was significantly increased by E2 in a dose-dependent fashion (Figure 10A), and the estrogen-mediated reduction of miR-26a expression was impaired in *Esr1*^−/−^ B cells, in the presence or absence of butyrate (Figure 10B). It has been suggested that in ER^+^ breast cancer cells, E2-ERα can indirectly reduce miR-26a expression by upregulating c-MYC, which has been reported to suppress the expression of many miRNAs (33, 43). This mechanism, however, may not play a significant role in E2-mediated miR-26a downregulation in B cells, as indicated by the unaltered *c-Myc* expression upon E2 treatment in CH12F3 B cells, in which miR-26a was inhibited by E2 and unaltered or even reduced *c-Myc* expression upon treatment of different doses of E2 in primary B cells (Figures 7D, 10C,D). Thus, estrogen reduces the expression of miR-26a, a silencer of *Aicda*, likely through both the classical and the non-classical ERα-signaling pathways.

## Discussion

Estrogen plays an important role in the generation of class-switched and mutated antibodies and autoantibodies, thereby contributing to the greater response to microbial vaccines as well as the greater incidence of antibody-mediated autoimmunity, such as in systemic lupus erythematosus, in females (4, 6–16, 28). Indeed, we have shown that estrogen enhances AID expression by activating the *HOXC4*/*HoxC4* promoter and inducing HoxC4, which is a critical *Aicda* promoter activator together with NF-κB (27, 28). *HOXC4*/*HoxC4* activation by estrogen-ER complexes occurs by binding to three evolutionarily conserved and cooperative EREs in the *HOXC4/HoxC4* promote (27, 28). As induced by CD40 or TLR and IL-4 signaling, HOXC4/HoxC4 synergizes with NF-κB to induce AID and CSR/SHM (27, 28). As we have shown, SCFA HDIs function as epigenetic modulators of T-dependent and T-independent class-switched and somatically hypermutated antibody responses as well as autoantibody responses by upregulating select miRNAs that target the 3′UTR of *Aicda* and *Prdm1* mRNAs (2, 23–25). Here, we demonstrated that estrogen reverses the SCFA HDI-mediated modulation of antibody and autoantibody responses (2, 23–25). This effect is mediated to a great extent by estrogen downregulation of miR-26a, which, as we showed, is upregulated by SCFA HDI and downregulates *Aicda* transcripts by targeting *Aicda* 3′UTR.

By binding to estrogen receptors ERα and/or ERβ, estrogen is involved in the regulation of multiple physiological processes. ERα and ERβ are ligand-inducible transcription factors that share common structural and functional domains and activate or repress gene expression in estrogen-responsive tissues (57). In addition to modulating many protein-coding genes, estrogen modulates expression of non-coding RNAs, including several miRNAs, such as miR-26a (31, 32). In the classical pathway of estrogen function, E2 diffuses into the cell and binds to ERs, which located mainly in the nucleus. After binding E2, ERs form dimers that bind to inverted palindromic ERE sequences in the regulatory regions of estrogen-responsive genes (57). In the non-classical pathway of estrogen-mediated gene expression, E2-ER-dependent regulation of gene expression occurs through protein-protein interaction of DNA-bound ER with other DNA-bound transcriptions factors, such as AP1 and Sp1 (49, 58). Indeed, transcription of many genes that depend on AP-1 can be repressed by ERs activated by E2 (52–55). In addition, E2 has also been suggested to repress gene transcription through ERα-Sp1, which recruits the transcriptional repressor-HDAC3 complex (56). The presence of multiple putative EREs, AP-1 motifs and Sp1-binding GC boxes in the promoter regions of miR-26a host genes *CTDSPL* and *CTDSP1* suggests that the E2-mediated regulation of miR-26a expression involves both classical and non-classical ER-action pathways possible resulting in overall net inhibition effect of miR-26a expression. c-Myc would provide an additional mechanism for E2-mediated downregulation of miR-26a. In ER^+^ breast cancer cells, E2-ERα can indirectly reduce miR-26a expression by upregulating c-MYC, which has been reported to inhibit the expression of several miRNAs (33, 43). Estrogen has been shown to induce *c-Myc* expression through an upstream enhancer activated by ER and AP-1 (59), and c-MYC can regulate the expression of miR-26a by directly binding to the promoters of miR-26a host genes (*CTDSPL* and *TDSP1*). However, this mechanism may not play a major role in E2-mediated miR-26a downregulation in B cells, as indicated by the unaltered or even reduced *c-Myc* expression upon E2 treatment of such cells. In addition to positive regulation of *c-Myc* through ERα-mediated activation of *c-Myc* promoter and enhancer regions, estrogen also promotes a negative regulatory pathway of *c-Myc* expression through miRNAs, thereby making the overall effect of estrogen on *c-Myc* expression a result of the balance of its positive and negative effects. Tipping such a balance would depend on the dose of estrogen. The E2 amount used in this study possibly promoted a stronger negative than positive effect on the regulation of *c-Myc* in primary mouse B cells.

Males and females display differential immunological responses to foreign- and self-antigens (7, 12, 60). Antibody responses to infections as well as bacterial and viral vaccines, such as influenza, hepatitis A, hepatitis B, rubella, measles, diphtheria, tetanus, *Brucella*, and rabies vaccines, are generally more vigorous in females than males (5, 61–75). Consistent with these differences, females show lower incidence of clinical disease than males after vaccination with influenza (76–79), hepatitis A (80, 81), and hepatitis B (82) vaccines. In addition, women vaccinated with half a dose of influenza vaccine achieved comparable antibody titers to males vaccinated with full dose (83). Genes on the X chromosome regulate immune function and play an important role in modulating sex differences in immune responses, and sex hormones interact with genetic and environmental factors to control immune responses (12, 84). In general, estrogen displays an immune enhancing activity whereas testosterone, the primary male sex hormone, has a suppressive effect on the immune system. Testosterone has recently been shown to enhance the association of GPR174, an X chromosome-encoded G-protein coupled receptor, with Gai in response to its ligand CCL21 and suppress B cell positioning toward the follicular center and germinal center formation (85). This may lead to a weaker antibody response in males. The effect of estrogen on the immune system can differ dependent on the level of estrogens, cell type, cell activation state, microenvironment, and the experimental context (9). Estrogen can have profound effects on B cell differentiation, activity, function, and survival (7, 9, 17). Our results further extend our previous findings and further emphasize an important role for estrogen in the regulation of AID expression and, therefore, the maturation of the antibody response.

Sex differences in immune responses also result in differential susceptibility of males and females to autoimmune diseases. A majority of autoimmune diseases, such as systemic lupus erythematosus, Sjögren syndrome, thyroid diseases, scleroderma, and myasthenia gravis, occur predominantly in females (12). Autoantibody response and autoimmune diseases are regulated by genetic, epigenetic, infectious factors, as well as external and internal environmental triggers, such as hormones and the microbiome. It is well-accepted that estrogens contribute to the immunopathogenesis of lupus, based on the tendency for disease onset in women during the child-bearing years, as well as increased relative risk of SLE onset and disease flares in women who were administrated exogenous estrogens (86). Many SLE patients show more symptoms before menstrual periods and/or during pregnancy, when estrogen incretion is high. Further, patients with Klinefelter's syndrome (XXY) characterized by hypergonadotrophic hypogonadism are prone to develop SLE (87). Finally, male-to-female transgenders have been reported to develop SLE after a sexual-reassignment operation and long-term treatment with exogenous estrogens (88–90).

Estrogen plays an important physiologic role in B cell development and function (4, 17). E2 can induce a genetic program that alters B cell survival and activation in a B cell intrinsic fashion and thus skews the naïve immune system toward autoreactivity (3). E2 has been shown to enhance B cell differentiation, increase the numbers of autoantibody-producing cells and overall autoantibody production (91, 92). E2 enhances anti-ds DNA antibody and IgG, IgM production by PBMCs from SLE patients (93). In lupus-prone NZB/WF1 mice, E2 administration resulted in an increased level of anti-C1q and anti-dsDNA autoantibodies, leading to accelerated glomerulonephritis and disease progression (94). Conversely, ERα deficiency in NZB/WF1 mice reduced anti-dsDNA IgG autoantibodies, attenuated glomerulonephritis, and increased survival (95). Progesterone, which in general has effect opposite to estrogen, reduced pathogenic anti-dsDNA IgG2a and glomerular IgG accumulation (96).

We have shown that HDIs upregulate the expression of select miRNAs that inhibit *Aicda* or *Prdm1* by increasing histone acetylation of the host genes of such miRNAs (23, 24). Two RNase III proteins, Drosha and Dicer are critical for miRNA biogenesis (97). Multiple acetyltransferases are involved in acetylating Drosha proteins, thereby preventing their degradation by inhibiting ubiquitination. For instance, acetylation of Drosha on the N-terminus inhibits Drosha degradation by ubiquitination, thereby increasing the stability of this protein (98). Drosha acetylation can be increased by HDI. Therefore, SCFA HDIs can increase Drosha protein level by enhancing its stability and, thereby, enhancing miRNA biogenesis. Conversely Drosha-mediated miRNA processing can be inhibited by ERα (99). This would provide a further potential mechanism by which HDIs upregulate miRNA expression, particularly miR-26a, and how estrogen downregulate this miRNA.

HDIs, including SCFAs butyrate and propionate, reduce both *Aicda* and *Prdm1* expression (2, 23–26). In the current studies, estrogen reversed HDI-mediated inhibition of *Aicda* expression, but did not significantly reverse HDI-induced reduction of *Prdm1* expression and plasma cell differentiation. *Prdm1* expression and plasma cell differentiation entail, among other modifications, DNA hypomethylation (100). DNA methylation is mediated by DNA methyltransferases (DNMTs), which catalyze the transfer of a methyl-group to DNA, and Tet dioxygenases, which oxidates 5-methylcytosine in DNA to 5-hydroxylmethylcytosine (5hmC), thereby mediating active DNA demethylation (101). Interestingly, miR-26a has been shown to modulate the expression of Tet2 (102, 103), the most abundant Tet in B cells (our unpublished data). Estrogen-mediated reduction of miR-26a would have the potential to upregulate Tet2, thereby leading to increased *Prdm1* expression and plasma cell differentiation (104). However, as miR-26a is also predicted to target *DNMT3B* (105), the possible activation of *DNMT3B* upon miR-26a reduction by estrogen might have overridden a potential active DNA demethylation caused by an increase in Tet2 expression. It is also possible that estrogen, through ERα, enhances expression of Ets1 (106), a negative regulator of *Prdm1*, thereby overriding the potentially activating effect of estrogen-ER on plasma cell differentiation. Finally, in addition to *Aicda*, miR-26a has been shown to target *Pten* (phosphatase and tensin homolog) gene (107), a lipid phosphastase, which directly antagonizes PI3K activity by dephosphorylating PIP_3_ to regenerate PI (4, 5) P_2_. B cell-specific *Pten* deficiency results in impaired CSR and enhanced plasma cell differentiation (108, 109). It is possible that the estrogen-mediated reduction of miR-26a in B cells also resulted in an upregulation of *Pten*, which might also contribute to CSR enhancement by estrogen.

SCFAs, such as butyrate and propionate, generated by gut commensal bacteria through digestion of dietary fibers, are among the most abundant endogenous HDIs in the body. The gut microbiota is a critical regulator of homeostasis by protecting against pathogens while maintaining immune tolerance to allergens. Commensal bacteria can modulate host immune responses through metabolite-dependent mechanisms (110). Butyrate and propionate are among the most abundant commensal bacterial metabolites with immunoregulatory functions. These endogenous SCFA HDIs play an important role in preventing or dampening dysregulated antibody responses, which can lead to generation of autoantibodies or allergic IgE, while maintaining a certain level of protective antibodies against microbial pathogens (25, 111, 112). Recent studies have suggested an important interaction between estrogen and gut microbiota (113, 114), which would act together to regulate immune responses. Gut commensal bacteria can be influenced by sex hormones including E2, which has been suggested to impact bacterial community composition (114). As we showed here, E2 can also modulate the effect of SCFAs, the major metabolites of gut commensal bacteria. The gastrointestinal microbiome profoundly impacts on various immune responses, including B cell maturation, activation and IgA antibody responses (115, 116). In the gut, which contains high concentration of butyrate and propionate, IgA is produced extensively. IgA can bind to the mucus layer covering epithelial cells and form a barrier capable of neutralizing pathogens before they reach the epithelial cells. By opposing the SCFA HDI-mediated downregulation of the antibody response, estrogen may be important for ensuring a physiologic high level of IgA in the gut. Indeed, the intestine is one of the major locations of estrogen metabolism and the intestine maintains a relatively high concentration of estrogen, including E2 (117, 118). Conversely, by opposing the SCFA HDI-mediated inhibition of class-switched autoantibody response, high level of estrogen, such as that in pre-ovulation women, may lift the homeostatic protection of gut microbiota from autoantibody production and autoimmunity. Thus, by showing that estrogen reverses the SCFA HDI-mediated downregulation of AID expression and CSR through selective modulation of miR-26a, our finding suggests that the “safeguard” function of SCFAs in dampening antibody overproduction and preventing emergence of autoantibodies can be reversed by estrogen. This may provide an explanation for the female bias in autoantibody-mediated autoimmune diseases, such as lupus, and the greatly increased incidence of food allergies in female adults (119).

## Data Availability Statement

All datasets generated for this study are included in the article/Supplementary Material.

## Ethics Statement

The animal study was reviewed and approved by the Institutional Animal Care and Use Committees of the University of Texas Health Science Center, San Antonio.

## Author Contributions

PC conceived and designed the study, planned the experiments, analyzed all primary data, created all figures, and wrote the manuscript. TS, YX, ZQ, JI, DC, and ZX performed experiments. HZ designed the study, planned, analyzed data, created all figures, and wrote the manuscript.

### Conflict of Interest

The authors declare that the research was conducted in the absence of any commercial or financial relationships that could be construed as a potential conflict of interest.

## Abbreviations

AID: activation-induced cytidine deaminase
CSR: class switch DNA recombination
ER: estrogen receptor
ERE: estrogen response element
GC: germinal center
HDI: histone deacetylase inhibitor
*IgH*: immunoglobulin heavy chain locus
miRNA: microRNA
NP-CGG: 4-hydroxy-3-nitrophenyl acetyl-conjugated chicken γ-globulin
SHM: somatic hypermutation.
