# Supplementary material for: Estrogen Reverses HDAC Inhibitor-Mediated Repression of Aicda and Class-Switching in Antibody and Autoantibody Responses by Downregulation of miR-26a
Source: Front Immunol. 2020 Mar 24;11:491. doi: 10.3389/fimmu.2020.00491 (PMC7105609; doi:10.3389/fimmu.2020.00491)
Supplement: Supplementary file 1 [file Data_Sheet_1.PDF]

Supplemental Table 1. Primers used for this study.

	Forward primer	Reverse primer
Mouse genes		
Aicda	5'-AGAAAGTCACGCTGGAGACC-3'	5'-CTCCTCTTCACCACGTAGCA-3'
Prdm1	5’-GCTGCTGGGCTGCCTTTGGA-3’	5’-GGAGAGGAGGCCGTTCCCCA-3’
cMyc	5’- CAGCTCGCCCAAATCCTGTA-3’	5’- GTGTCTCCTCATGCAGCACT-3’
Gapdh	5′-TTCACCACCATGGAGAAGGC-3′	5′-GGCATGGACTGTGGTCATGA-3′
Post-recombination transcripts		
Iμ-Cγ1	5’-ACCTGGGAATGTATGGTTGTGGCTT-3’	5′-ATGGAGTTAGTTTGGGCAGCA-3′
Iμ-Cε	5’-ACCTGGGAATGTATGGTTGTGGCTT-3’	5’-ACAGGGCTTCAAGGGGTAGA-3’
Mature miRNA and sn/snoRNA forward primers (used with Qiagen miScript Universal Primer as reverse primer)		
miR-155-5p	5’-TTAATGCTAATTGTGATAGG-3’	
miR-182-5p	5'-TTTGGCAATGGTAGAACTCACACCG-3'	
miR-361-5p	5’-TTATCAGAATCTCCAGGGGTAC-3’	
miR-92b-3p	5’-TATTGCACTCGTCCCGGCCTCC-3’	
miR-125a-5p	5'-TCCCTGAGACCCTTTAACCTGTGA-3'	
miR-26a-5p	5′-TTCAAGTAATCCAGGATAGGCT-3′	
Rnu6/RNU6-1/2	5’-GCTTCGGCAGCACATATACTAAAAT-3’	
Snord61/SNORD61	5’-CCACTGATCTTCCGACATGA-3’	
Snord68/SNORD68	5’-GCTGTACTGACTTGATGA-3’	
Snord70/SNORD70	5’-TTTTGGAACTGAATCTAAGTGATTT-3’	
CTDSPL and *CTDSP1* promoter ChIP		
CTDSPL promoter	5’-TACCCCAACTCTATGCCCGA-3’	5’-TTGCAGTCGGTAGTGGGTTC-3’
CTDSP1 promoter	5’-ATCGTTCCAAGGATCCCGAG-3’	5’-CTTCCCAGCAGATAGCGGG-3’
